# Comparison of Efficacy and Safety of Different Medication Protocols in Patients with Immunoglobulin G4–Related Disease Based on Follow-up Time: A Systematic Review and Network Meta-analysis

**DOI:** 10.5152/ArchRheumatol.2026.25136

**Published:** 2026-01-16

**Authors:** Yanwen Liu, Xianghui Fu, Youqun Zhang, Yan Zheng, Junfeng Jia, Zhaohui Zheng, Ping Zhu, Kui Zhang

**Affiliations:** Department of Clinical Immunology, Xijing Hospital, Fourth Military Medical University, Xi'an, China

**Keywords:** DMARDs, follow-up time, glucocorticoids, IgG4-related disease, network meta-analysis

## Abstract

**Background/Aims:**

Glucocorticoids (GCs) and disease-modifying antirheumatic drugs (DMARDs) are commonly used drugs in the treatment of immunoglobulin G4–related disease (IgG4-RD). However, no broad consensus is available on their intervention effects. Therefore, the efficacy and safety of different medication protocols in the treatment of IgG4-RD were assessed in this systematic review and network meta-analysis.

**Materials and Methods:**

This study was conducted following the Preferred Reporting Items for Systematic Reviews and Meta-Analyses. RStudio and Stata 15.1 were used for data analysis.

**Results:**

The results showed that in terms of improvement of remission rates, GCs + DMARDs had the strongest overall efficacy [surface under the cumulative ranking curve (SUCRA) = 82.9%], and DMARDs were the most effective within 12 months during follow-up (SUCRA = 82.5%), while GCs + DMARDs were the most effective over 12 months during follow-up (SUCRA = 83.2%). In terms of reduction of relapse rates, the overall efficacy of GCs + DMARDs was the strongest (SUCRA = 83.5%), and GCs + DMARDs performed the best both within and over 12 months during follow-up. The adverse reaction rates were 38.9%, 5.3%, and 33.3%, respectively, among patients treated with GCs + DMARDs, DMARDs, and GCs.

**Conclusion:**

The GCs + DMARDs are recommended for short-term improvement of remission rates and reduction of relapse rates, as well as for achieving long-term efficacy.

**Main points**

- First comprehensive network meta-analysis comparing the efficacy and safety of 4 treatment protocols (glucocorticoids (GCs), disease-modifying antirheumatic drugs (DMARDs), GCs + DMARDs, watchful waiting) for immunoglobulin G4-related disease (IgG4-RD) with stratified analysis by follow-up duration (≤/>12 months).
- Combination therapy superiority: The GCs + DMARDs demonstrated the strongest overall efficacy for improving remission rates surface under the cumulative ranking curve (SUCRA = 82.9%) and reducing relapse rates (SUCRA = 83.5%), outperforming monotherapies and watchful waiting across all time frames.
- Safety profile: The DMARDs had the lowest adverse reaction rate (5.3%), significantly lower than GCs (33.3%) and GCs + DMARDs (38.9%). Common adverse events included infections, glucose intolerance, and gastrointestinal reactions.
- Clinical recommendation: The GCs + DMARDs is recommended as the preferred regimen for both short-term disease control and sustained long-term management of IgG4-RD, balancing efficacy with manageable toxicity.

## Introduction

Immunoglobulin G4 (IgG4)–related disease (IgG4-RD), an immune-mediated systemic fibroinflammatory disease, is characterized by active multi-organ lesions, IgG4-positive plasma cell infiltration, and elevated serum IgG4 concentrations.^1^^-^^3^ Patients with IgG4-RD usually present with enlargement and fibrosis of a single or multiple organs or tissues, including the pancreas, bile ducts, salivary glands, retroperitoneum, sinuses, and orbits,^4^^,^^5^ which can lead to symptoms of obstruction or compression and irreversible organ damage. Therefore, early identification and prompt appropriate treatment of IgG4-RD are critical, and tumor-like lesions or even organ failure may occur if no prompt and effective treatment is available,^6^ greatly affecting the quality of life and longevity of patients.

Glucocorticoids (GCs) and disease-modifying antirheumatic drugs (DMARDs) are the commonly used drugs in the treatment of IgG4-RD nowadays. In particular, GCs can rapidly ameliorate clinical symptoms and reduce levels of blood biochemical indicators since most patients are sensitive to steroid therapy, but up to 40% of patients experience a relapse within the first year post-treatment.^7^ In addition, long-term use of GCs may lead to a series of adverse reactions, such as infection, glucose intolerance, and osteoporosis.^8^ Increasingly more novel DMARDs have been applied in the treatment of IgG4-RD in recent years, such as abatacept and iguratimod,^9^^,^^10^ achieving favorable effects. Different conclusions were made in several studies regarding the efficacy of GCs + DMARDs vs. GCs on IgG4-RD. Some studies held that GCs + DMARDs are superior to GCs in controlling the condition and reducing relapse,^11^^,^^12^ while some showed no difference in the relapse rate between the 2 medication protocols.^13^^,^^14^

In addition, most studies disagree on which medication protocol is the most effective at different follow-up times. Research suggested that the remission rate is higher when DMARDs are used than when GCs or GCs + DMARDs are used at 6 months during follow-up,^15^ while others showed that DMARDs produce a lower remission rate than GCs over 5 years during follow-up.^16^ Therefore, a network meta-analysis (NMA) was carried out on 4 different medication protocols (GCs, DMARDs, GCs + DMARDs, and watchful waiting) to compare their efficacy in the treatment of IgG4-RD, and subgroup analyses were performed based on different follow-up times, thereby clarifying the efficacy and safety of each protocol.

## Methods

### Study Registration

This NMA was conducted fully following the Preferred Reporting Items for Systematic Reviews and Meta-Analyses (PRISMA)^17^ to improve the quality of reporting of meta-analyses, and the PRISMA checklist is available in Supplementary Table 1. The study protocol had been registered with PROSPERO (CRD42024521672).

### Literature Search Strategy

A search was carried out in 4 English databases (PubMed, Embase, Web of Science, and Cochrane Library) in this NMA from inception to December 25, 2024. Medical subject headings plus text words were used in the search, including “IgG4-related disease,” “glucocorticoids,” “immunosuppressants,” “biologics,” “disease-modifying antirheumatic drugs,” “rituximab,” “retrospective studies,” “prospective studies,” “cohort studies,” and “case-control studies.” The search strategy was modified according to the database characteristics and Participant, Intervention, Comparison, Outcome, and Study (PICOS) principle.^18^ A manual search was also conducted to identify potentially missing relevant studies. The complete search method is described in Supplementary Table 2.

### Eligibility Criteria

All included studies followed the PICOS principle:

Participant: 1) Patients diagnosed with IgG4-RD based on the recognized diagnostic criteria at the time of publication, regardless of gender, race, ethnicity, duration of disease, and etiology; 2) Patient age ≥14 years.

Intervention: GCs, DMARDs, or GCs + DMARDs were given to the patients in the intervention group. The duration of treatment was at least 3 months. The DMARDs included conventional DMARDs (cDMARDs) and biological DMARDs (bDMARDs), the former of which included drugs such as mycophenolate mofetil, cyclophosphamide, and methotrexate, and the latter of which included drugs such as rituximab (RTX) and abatacept.

Comparison: Only watchful waiting was adopted in the control group. Watchful waiting was defined as temporarily performing no immediate treatment after IgG4-RD diagnosis but observing disease progression through regular monitoring and follow-up.

Outcome: 1) Remission rate. Disease remission was defined as improvement in related symptoms of IgG4-RD, reduction in the number of involved lesions, or a decrease in the IgG4-RD response index of ≥2 from baseline;^19^^,^^20^ 2) Relapse rate. Disease relapse was defined as recurrence of enlargement of involved organs and/or worsening of imaging findings, with or without a re-increase in serum IgG4 levels;^21^ 3) Adverse reactions.

Study: Observational studies. Minimum sample size ≥5.

Exclusion criteria: 1) Meta-analyses, reviews, or guidelines; 2) conference papers, replies, or comments; 3) case reports; 4) animal or in vitro experimental studies; 5) studies published in a language other than English; 6) duplicate publications; 7) incorrect or incomplete data; and 8) unavailable full text.

### Data Extraction

After duplicate publications were eliminated using EndNote20, 2 reviewers were independently responsible for screening titles and abstracts from the search results, and then conducting a full-text search to identify eligible studies based on the inclusion and exclusion criteria. In case of disagreement, they could discuss or consult a third reviewer for resolution. A standardized data collection form was used for data extraction, including main author, country, and year of publication of the included studies, sample size, mean age, and clinical characteristics of the participants, treatment protocols for IgG4-RD, duration of follow-up, remission and relapse rates, and all adverse reactions (infections, osteoporosis, diabetes mellitus, hypertension, and hepatic dysfunction) in each group.

### Quality Assessment

The quality assessment of the included studies was performed by 2 reviewers using the Newcastle–Ottawa Scale (NOS),^22^ covering study population selection, comparability, and outcome. The semi-quantitative principle of a star scale was used with a maximum of 9 stars, and each item was assessed at a maximum of 1 star, except for comparability which was assessed at a maximum of 2 stars. A higher score suggested a higher quality of study. Any disagreements were resolved by discussion with a third reviewer.

### Synthesis Methods

Bayesian NMA was carried out, and the Stata 15.1 (StataCorp LLC; College Station, TX, USA) “network” command and the RStudio 4.3.2 (Posit PBC; Boston, MA, USA) “gemtc” and “coda” packages were used for statistical analyses. The Markov Chain Monte Carlo method was utilized for modeling, with 4 simultaneous Markov chains running and the number of iterations set to 20 000, and the modeling was completed after 50 000 simulation iterations. For dichotomous variables, relative risk (RR) was used as the effect size index, along with a 95% CI, and the difference was considered statistically significant between 2 groups when the 95% CI of RR did not contain 1. Whether the overall consistency and inconsistency of the model fit were uniform was compared by the deviance information criterion (DIC). The DIC difference <5 was deemed uniform between overall consistency and inconsistency, and vice versa. The nodal split method was used for further local consistency testing in the case of a closed loop, and *P* < .05 suggested local inconsistency in 2 interventions. Heterogeneity was assessed using the *I^2^* statistic, and the estimated *I^2^* >50% suggested high heterogeneity. In addition, the interventions were ranked based on the surface under the cumulative ranking curve (SUCRA), and the differences in their efficacy were displayed using a rankogram. The closer the SUCRA value was to 100%, the higher the likelihood that the intervention was the optimal protocol. Publication bias tests were performed using the funnel plot on the results that included more than 10 studies. Subgroup analyses were also conducted on the relapse and remission rates when different medication protocols were adopted at different follow-up time.

## Results

### Study Selection

**Figure 1.**
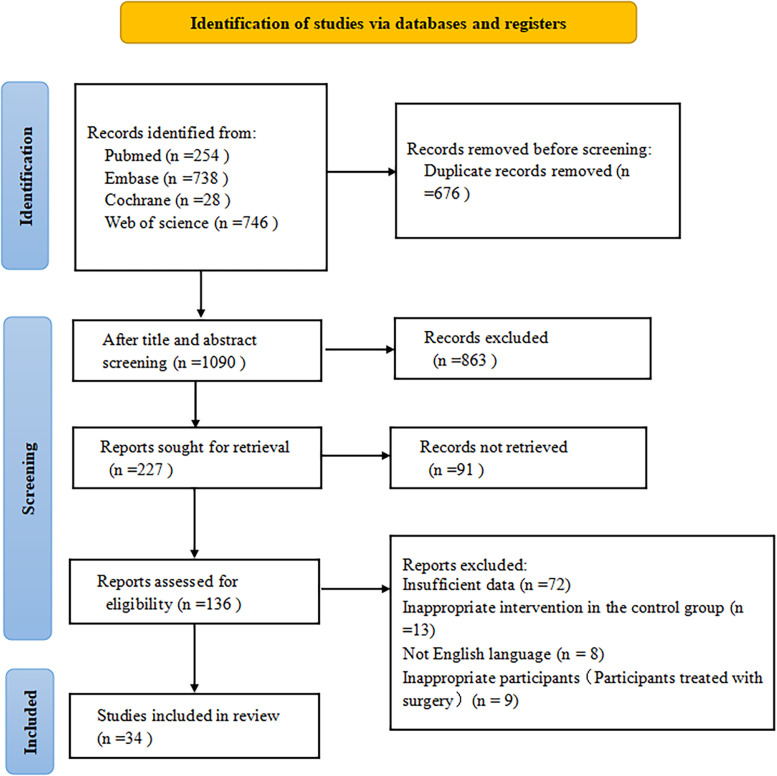
Literature screening procedure.

Initially, 1766 records were retrieved using the literature search strategy. After 676 duplicates were deleted, 1090 records were obtained, of which 863 were excluded based on the title and abstract. After the full-text search, 34 eligible studies were acquired finally,^6^^,^^9^^,^^11^^,^^12^^,^^14^^-^^16^^,^^19^^,^^23^^-^^48^ and were included in this study, all of which had been published. The literature screening procedure is described in Figure 1.

### Study Characteristics

In the 34 observational studies included, 4337 patients underwent one of the 4 treatments for IgG4-RD. These studies were from Asia (n = 21), North America (n = 7), and Europe (n = 6). The duration of follow-up was 3-240 months. All studies were published in English from 2008 to 2023. The characteristics of the included studies are shown in Supplementary Table 3.

### Quality Assessment Results

The quality of the included studies was evaluated using the NOS and assessed the selection bias of each included study from the “Selection,” “Comparability,” and “Outcome” domains. The highest score in the “Selection” domain was 4, and all included studies were scored ≥3; the highest score in the “Comparability” domain was 2, and 16 out of the 34 included studies were scored 2; the highest score in the “Outcome” domain was 3, and all included studies were scored ≥2. The results of the quality assessment of the included studies using NOS revealed that all studies had high methodological quality, with 13 studies scoring 7, 14 studies scoring 8, and 7 studies scoring 9. The risk of bias for the included studies is displayed in Supplementary Table 3.

### Meta-Analysis

#### Remission:

**Figure 2.**
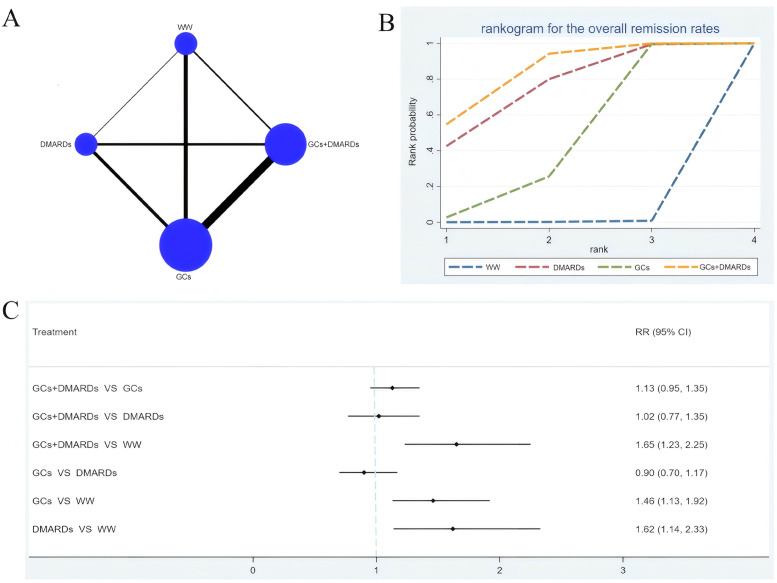
Network meta-analysis results for the overall remission rates. (A): Network diagram. Each node represents 1 medication protocol, and its size is proportional to the total number of patients receiving this medication protocol. Each line represents a head-to-head comparison, and its width is proportional to the number of studies comparing the connected protocols. (B): Cumulative probability ranking curve of different interventions; different interventions are ranked based on the surface under the cumulative ranking curve (SUCRA). The closer the SUCRA value is to 100%, the higher the likelihood that the intervention is the optimal protocol. (C): Forest plot. GCs, glucocorticoids; DMARDs, disease-modifying antirheumatic drugs; WW, Watchful waiting.

Seventeen studies involving 2749 patients were included.^9^^,^^11^^,^^12^^,^^15^^,^^16^^,^^19^^,^^23^^,^^28^^,^^29^^,^^32^^,^^37^^,^^43^^-^^46^^,^^48^ The network diagram comparing the overall remission rates among medication protocols is shown in Figure 2A. The network diagram comparing the remission rates within 12 months (included) during follow-up and the network diagram comparing the remission rates over 12 months during follow-up are shown in Supplementary Figure 1A, 2A, respectively.

It was found by the meta-analyses on the overall remission rates of different medication protocols that GCs + DMARDs (RR = 1.65, 95% CI: 1.23-2.25), GCs (RR = 1.46, 95% CI: 1.13-1.92), and DMARDs (RR = 1.62, 95% CI: 1.14-2.33) all achieved higher remission rates than watchful waiting (Figure 2C). In the rankogram, GCs+DMARDs ranked first (SUCRA = 82.9%), followed by DMARDs (74.0%) and GCs (42.7%) (Figure 2B).

The results of meta-analyses on the remission rates within 12 months during follow-up revealed that the remission rates of GCs + DMARDs (RR = 1.94, 95% CI: 1.07-3.49) and DMARDs (RR = 2.09, 95% CI: 1.09-4.11) were higher than watchful waiting (Supplementary Figure 1C). In the rankogram, DMARDs ranked first (SUCRA = 82.5%), followed by GCs + DMARDs (73.3%) and GCs (42.2%) (Supplementary Figure 1B).

By the meta-analyses on the remission rates over 12 months during follow-up, no statistically significant difference was found in the pairwise comparison of medication protocols (*P* > .05) (Supplementary Figure 2C). In the rankogram, GCs + DMARDs ranked first (SUCRA = 83.2%), followed by DMARDs (54.8%) and GCs (53.0%) (Supplementary Figure 2B).

#### Relapse:

**Figure 3.**
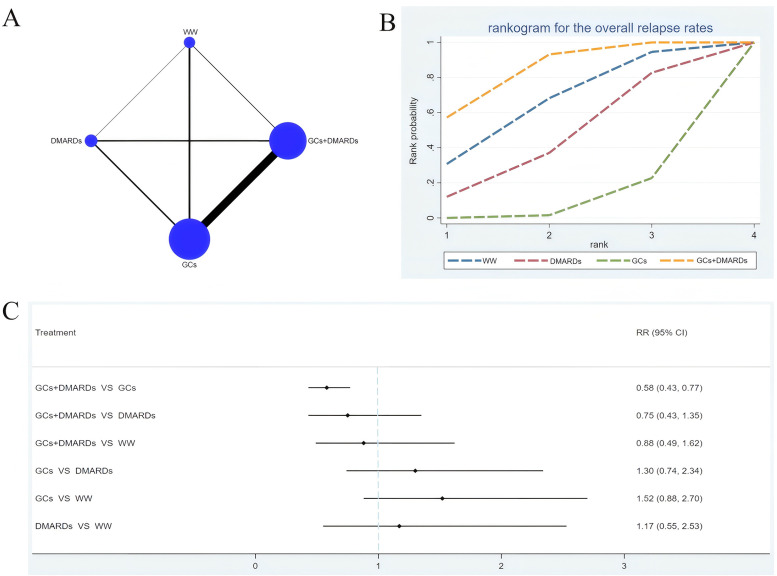
Network meta-analysis results for the overall relapse rates. (A): Network diagram; (B): Cumulative probability ranking curve of different interventions; (C): Forest plot. GCs, glucocorticoids; DMARDs, disease-modifying antirheumatic drugs; WW, Watchful waiting.

Twenty-seven studies involving 4106 patients were included.^11^^,^^12^^,^^14^^,^^15^^,^^23^^-^^48^ The network diagram comparing the overall relapse rates among medication protocols is shown in Figure 3A. The network diagram comparing the relapse rates within 12 months during follow-up and the network diagram comparing the relapse rates over 12 months during follow-up are shown in Supplementary Figure 3A, 4A, respectively.

It was found by the meta-analyses on the overall relapse rates of different medication protocols that GCs + DMARDs achieved lower relapse rates than GCs (RR = 0.58, 95% CI: 0.43-0.77) (Figure 3C). In the rankogram, GCs + DMARDs ranked first (SUCRA = 83.5%), followed by watchful waiting (64.5%) and DMARDs (43.9%) (Figure 3B).

The results of meta-analyses on the relapse rates within 12 months during follow-up revealed that the relapse rates of GCs + DMARDs were lower than GCs (RR = 0.49, 95% CI: 0.33-0.71) (Supplementary Figure 3C). In the rankogram, GCs + DMARDs ranked first (SUCRA = 91.7%), followed by DMARDs (48.8%) and GCs (9.5%) (Supplementary Figure 3B).

The results of meta-analyses on the relapse rates over 12 months during follow-up revealed that the relapse rates of GCs+DMARDs were lower than GCs (RR = 0.64, 95% CI: 0.41-0.99) (Supplementary Figure 4C). In the rankogram, GCs+DMARDs ranked first (SUCRA=74.4%), followed by watchful waiting (66.3%) and DMARDs (45.3%) (Supplementary Figure 4B).

#### Adverse Reactions:

Adverse reactions occurring during treatment with 3 different medication protocols were mentioned and detailed in 6 studies,^11^^,^^12^^,^^33^^,^^36^^,^^37^^,^^47^ including infections, gastrointestinal reactions, impaired hepatic function, and glucose intolerance (Table 1). The adverse reaction rates were 38.9%, 5.3%, and 33.3%, respectively, among patients treated with GCs + DMARDs, DMARDs, and GCs. The qualitative meta-analysis revealed that GCs-containing protocols (GCs and GCs + DMARDs) most frequently induced metabolic toxicity (e.g., glucose intolerance) and gastrointestinal toxicity. In contrast, hematologic events (e.g., leukopenia) were reported only for GCs + DMARDs (Supplementary Table 4). Despite limited data, these findings highlighted organ-specific risks, particularly for GCs-based protocols.

#### Publication Bias:

**Figure 4.**
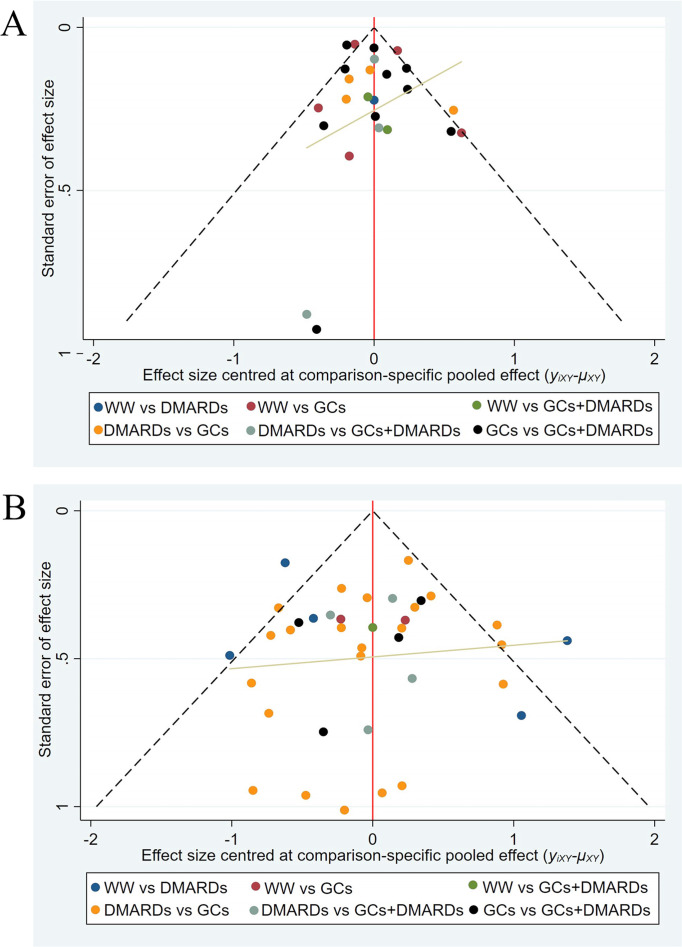
Funnel plots of outcomes. (A): the overall remission rates; (B): the overall relapse rates. GCs, glucocorticoids; DMARDs, disease-modifying antirheumatic drugs; WW, Watchful waiting.

More than 10 studies were included in the analysis of the overall remission and relapse rates, so the publication bias was assessed by funnel plots, with points of different colors indicating comparisons among the 4 medication protocols. As shown in the funnel plots, the remission rates displayed no publication bias (Figure 4A), whereas the yellow line segments of the relapse rates tended to be parallel to the X-axis, indicating possible publication bias (Figure 4B).

#### Sensitivity Analyses:

Network meta-analysis was carried out on the overall remission and relapse rates using random-effects and fixed-effects models, respectively, and the similarities in the total residual deviance (totresdev), penalty term for deviance (pD), and DIC values were observed. The results showed that the values of the 3 parameters were similar between the 2 models (overall remission rate: fixed-effects vs. random-effects: totresdev = 39.93 vs. 40.07, DIC = 69.35 vs. 67.84, pD = 29.42 vs. 27.77. Overall relapse rate: fixed-effects vs. random-effects: totresdev = 56.80 vs. 57.36, DIC = 103.52 vs. 102.46, pD = 46.72 vs. 45.10), suggesting good similarity in the included studies and stable and reliable data.

#### Subgroup Analyses:

To assess the impact of demographics on the results, subgroup analyses were performed by region. There were 21 studies including 2803 patients in Asia, 7 studies including 458 patients in North America, and 6 studies including 1076 patients in Europe. The number of studies in North America and Europe was too small to conduct an NMA, so both were combined as non-Asian studies. To determine the presence or absence of regional differences in the distribution of involved organs, organ involvement in the included studies was statistically analyzed. Sixteen studies reported single-organ involvement, while the remaining 18 studies reported systemic organ involvement specifically in the Asian cohort (10 studies) and non-Asian cohort (8 studies); the top 10 involved organs were largely the same in both cohorts (Supplementary Table 5). Both Asian studies (SUCRA = 80.4%) and non-Asian studies (SUCRA = 75.8%) revealed that GCs + DMARDs were most effective in improving the overall remission rate, and both Asian studies (SUCRA = 92.2%) and non-Asian studies (SUCRA = 92.9%) revealed that GCs + DMARDs performed best in reducing the overall relapse rate. The results of subgroup analyses were consistent with those of the original studies (Supplementary Table 6). Heterogeneity analyses showed that the overall *I*^2^ values for the remission and relapse rates were 46.3% and 33.3% in Asian studies, and 48.3% and 0% in non-Asian studies, respectively, all less than 50%, suggesting that the results were stable and reliable.

To assess the impact of different DMARDs on study results, subgroup analyses were conducted on bDMARDs and cDMARDs. The meta-analysis revealed that the overall remission rates of GCs + cDMARDs (RR = 1.72, 95% CI: 1.26-2.39), GCs alone (RR = 1.50, 95% CI: 1.14-2.00), and bDMARDs alone (RR = 2.01, 95% CI: 1.28-3.18) were all higher than that of watchful waiting (Supplementary Table 7). In the rankogram, bDMARDs alone ranked first (SUCRA = 88.8%), followed by GCs + cDMARDs (71.4%) and GCs + bDMARDs (58.0%). Besides, the overall relapse rate of GCs + cDMARDs was lower than that of GCs alone (RR = 0.50, 95% CI: 0.34-0.70) (Supplementary Table 7). In the rankogram, GCs + bDMARDs ranked first (SUCRA = 80.51%), followed by GCs + cDMARDs (65.0%) and bDMARDs alone (63.7%). Subgroup analyses on different DMARDs also revealed that GCs + bDMARDs or GCs + cDMARDs raised the remission rate and reduced the relapse rate.

## Discussion

This NMA included 34 observational studies involving 4337 IgG4-RD patients, and they were treated with GCs, DMARDs, GCs + DMARDs, or watchful waiting. This is the first study that comprehensively compared the efficacy and safety of the 4 medication protocols in the treatment of IgG4-RD within and over 12 months during follow-up. The results showed that in terms of improvement of remission rates, GCs + DMARDs had the strongest overall efficacy, and DMARDs had a higher efficacy within 12 months during follow-up, while GCs + DMARDs were the most effective over 12 months during follow-up. In terms of reduction of relapse rates, the overall efficacy of GCs + DMARDs was the strongest, and GCs + DMARDs performed the best both within and over 12 months during follow-up. Moreover, DMARDs had a lower incidence of adverse reactions in comparison with GCs and GC + DMARDs.

Currently, the treatment of IgG4-RD consists of remission induction and maintenance therapy stages. The former aims to reduce focal inflammation and rapidly relieve symptoms, while the latter aims to maintain disease remission. In this study, the remission rates of different medication protocols in the 2 stages were explored based on the follow-up time. It was found that GCs + DMARDs had the strongest overall efficacy in improving the remission rate. A meta-analysis showed that GCs plus immunosuppressants can achieve higher remission rates than GCs;^49^ multiple studies^50^^,^^51^ suggested that GCs plus RTX have a higher remission rate than RTX, consistent with the findings. Besides, DMARDs achieved the best effect within 12 months during follow-up. Rituximab was the major type of DMARDs in the included studies, and its ability to effectively treat IgG4-RD and rapidly relieve clinical symptoms has been verified.^52^ When used in the treatment of IgG4-RD, RTX can achieve a remission rate of more than 90%, and the remission rate has no increase when combined with GCs,^19^ which may contribute to the higher remission rate of DMARDs. In addition, DMARDs were mostly used in mild cases in the included studies, which may also account for the highest remission rate of DMARDs. In addition, GCs + DMARDs were the most effective over 12 months during follow-up. Immunosuppressants were the major type of DMARDs in the included studies. As reported by a meta-analysis covering 15 studies, GCs plus immunosuppressants achieve higher remission rates than GCs in the treatment of IgG4-RD,^53^ consistent with the findings in this paper.

The relapse rate of IgG4-RD increases with the extension of follow-up time,^40^ and the course of the disease exhibits a “relapse-remission” pattern,^54^ which is prone to repeated attacks if not properly treated. In this study, GCs + DMARDs produced lower relapse rates than GCs at different follow-up times. The conclusions regarding the relapse rates in different medication protocols vary across studies. Some studies held that GCs + DMARDs can reduce relapse compared with GCs,^33^ while some suggested that the relapse rate has no difference between the 2 protocols.^26^ This study demonstrated that GCs + DMARDs yielded a lower relapse rate than GCs. A randomized controlled clinical study revealed that GCs + DMARDs are superior to GCs in preventing relapse of IgG4-RD, consistent with the results in this paper.^55^ In the treatment of IgG4-RD, GCs work primarily by reducing inflammatory factors to relieve symptoms and are usually tapered off gradually due to many adverse reactions during long-term use, so relapse occurs easily.^56^^,^^57^ With a synergistic effect, GCs + DMARDs may contribute to the sustained control of tissue inflammation and reduce the relapse rate in IgG4-RD patients. Publication bias was indeed present in the assessment of relapse rates, which might result from the region of publication, the grade of periodicals, and sources of funding.

This NMA had several limitations. First, the sample size was small in some results, and differences were present in the dosage of GCs and patient baseline data, so larger-scale, high-quality randomized controlled studies are required to enhance the quality of evidence in the future. Second, only English-language studies were included. Moreover, the risk of confounding bias introduced by observational studies and the between-study heterogeneity in diagnostic criteria posed significant challenges to internal validity and inter-study comparability, thus increasing uncertainty in efficacy and safety estimates and weakening the reliability of comparison between different medication protocols. Random-effects models and sensitivity analyses were employed to mitigate these issues, but the inherent limitations of these methodologies cannot be fully eliminated. Therefore, the results of this NMA should be regarded as the best estimate under the current evidence base, and the conclusions should be interpreted with caution. In the future, high-quality randomized controlled trials (RCTs) with standardized diagnostic criteria are required for validation. Finally, due to the limited reporting quality of the original studies, only 1 study was included for the adverse reactions of DMARDs alone, with a small sample size, so the results may not be robust. Meanwhile, none of the included studies reported renal events, indicating gaps in safety monitoring. Therefore, future studies should prioritize prospective safety monitoring.

In conclusion, this NMA demonstrated that GCs + DMARDs can maintain a higher remission rate and the lowest relapse rate than other medication protocols. The GCs + DMARDs are recommended for both short-term and long-term medication. Currently, increasingly more treatment options for IgG4-RD are available, and individualized and precise treatment is the direction of development in this field. Large-scale RCTs are needed in the future to validate the relative efficacy of different medication protocols. Given the great heterogeneity in the natural course of IgG4-RD across different involved organs, organ-specific clinical trials or registry studies are also required to provide detailed data for individualized treatment strategies.

## Supplementary Materials

Supplementary Material

## Figures and Tables

**Table 1.** The Occurrence of Adverse Reactions of 3 Different Medication Protocols During Treatment

Medication Protocol	GCs + DMARDs	DMARDs	GCs
Number of included studies	6	1	5
Sample size	285	19	252
Infections	43	1	38
Glucose intolerance	31	0	25
Gastrointestinal reactions	23	0	12
Impaired hepatic function	6	0	6
Hypertension	2	0	3
Leukopenia	5	0	0
Myelosuppression	1	0	0
Total probability, %	38.9	5.3	33.3

## Data Availability

The data that support the findings of this study are available on request from the corresponding author.
