# Supplementary material for: Comparison of Efficacy and Safety of Different Medication Protocols in Patients with Immunoglobulin G4–Related Disease Based on Follow-up Time: A Systematic Review and Network Meta-analysis
Source: Arch Rheumatol. 2026 Jan 16;41(1):3–13. doi: 10.5152/ArchRheumatol.2026.25136 (PMC12869723; doi:10.5152/ArchRheumatol.2026.25136)
Supplement: Supplementary Material [file supplementary_material.pdf]

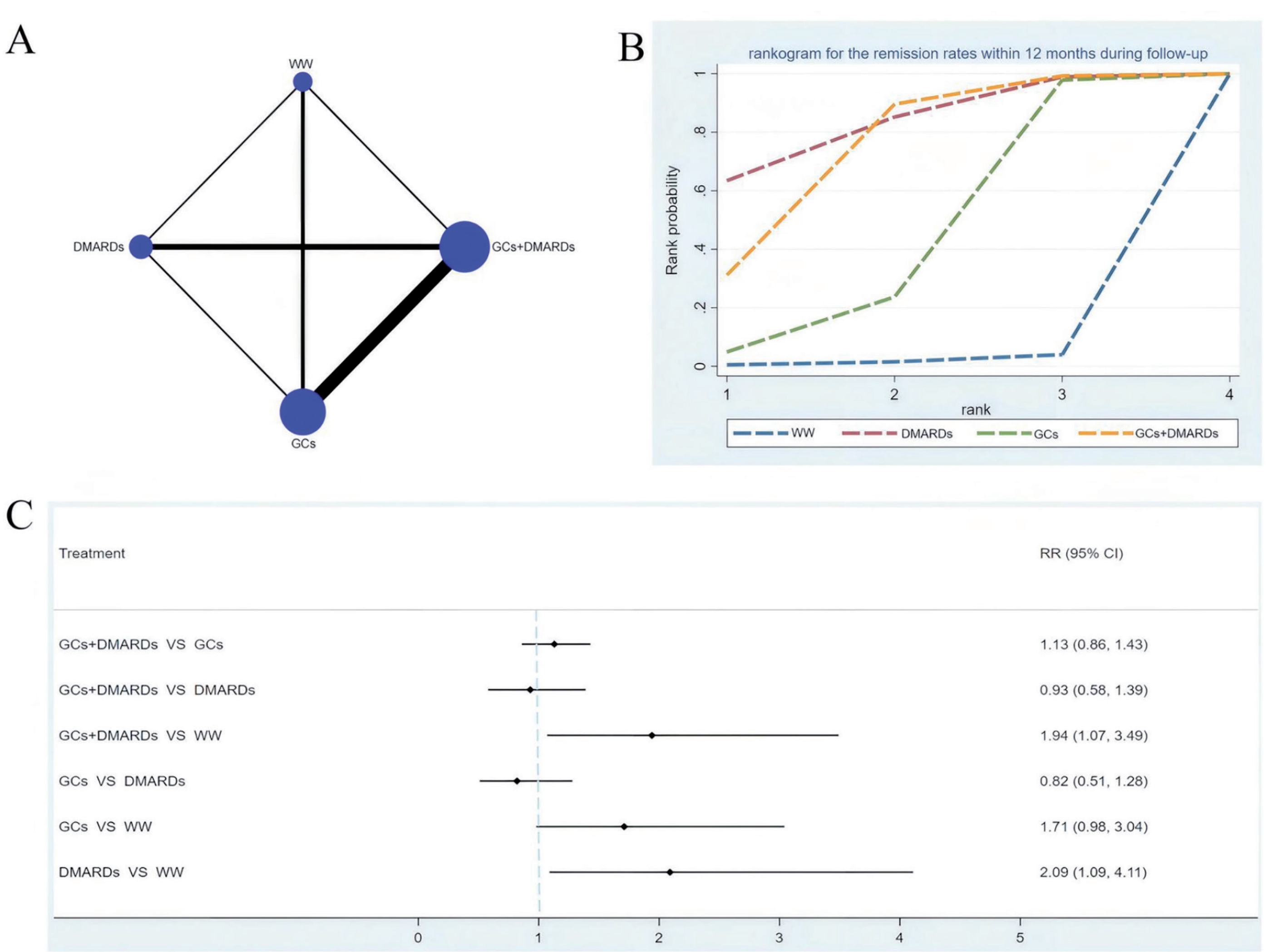

Supplementary Figure 1. Network meta-analysis results for the remission rates within 12 months. (A): Network diagram; (B): Cumulative probability ranking curve of different interventions; (C): Forest plot. GCs, glucocorticoids; DMARDs, disease-modifying antirheumatic drugs; WW, Watchful waiting.

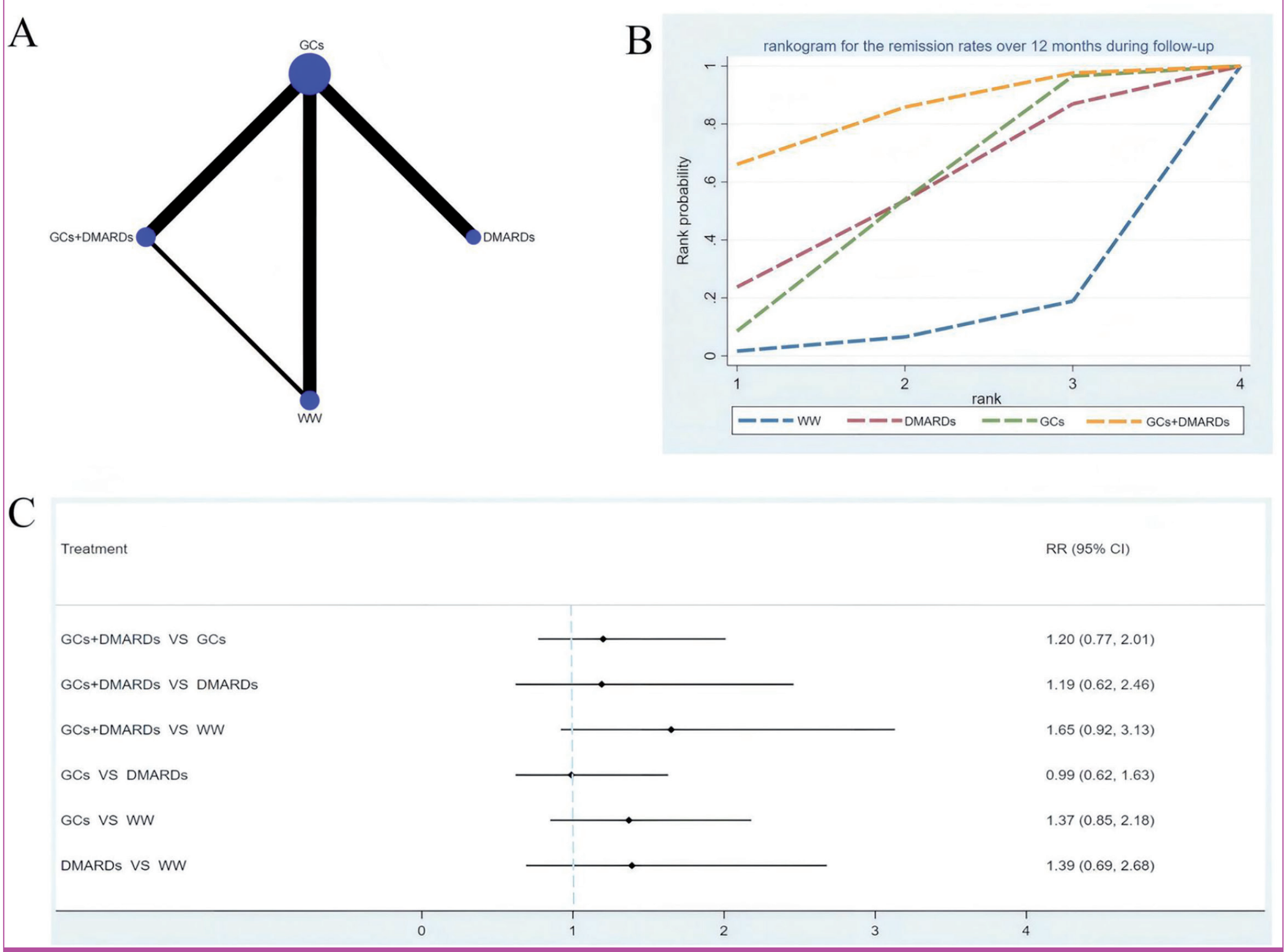

Supplementary Figure 2. Network meta-analysis results for the remission rates over 12 months. (A): Network diagram; (B): Cumulative probability ranking curve of different interventions; (C): Forest plot. GCs, glucocorticoids; DMARDs, disease-modifying antirheumatic drugs; WW, Watchful waiting.

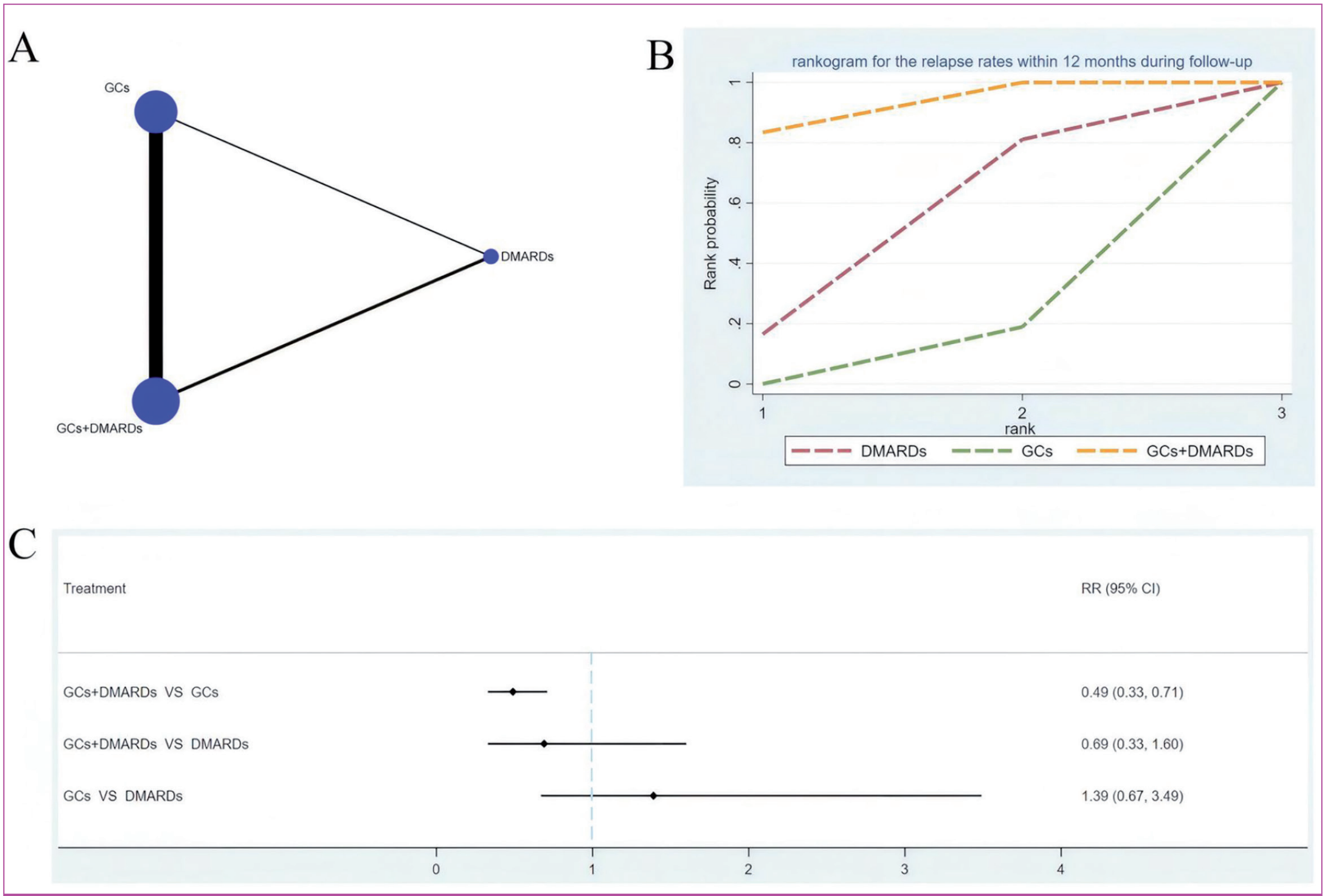

Supplementary Figure 3. Network meta-analysis results for the relapse rates within 12 months. (A): Network diagram; (B): Cumulative probability ranking curve of different interventions; (C): Forest plot. GCs, glucocorticoids; DMARDs, disease-modifying antirheumatic drugs; WW, Watchful waiting.

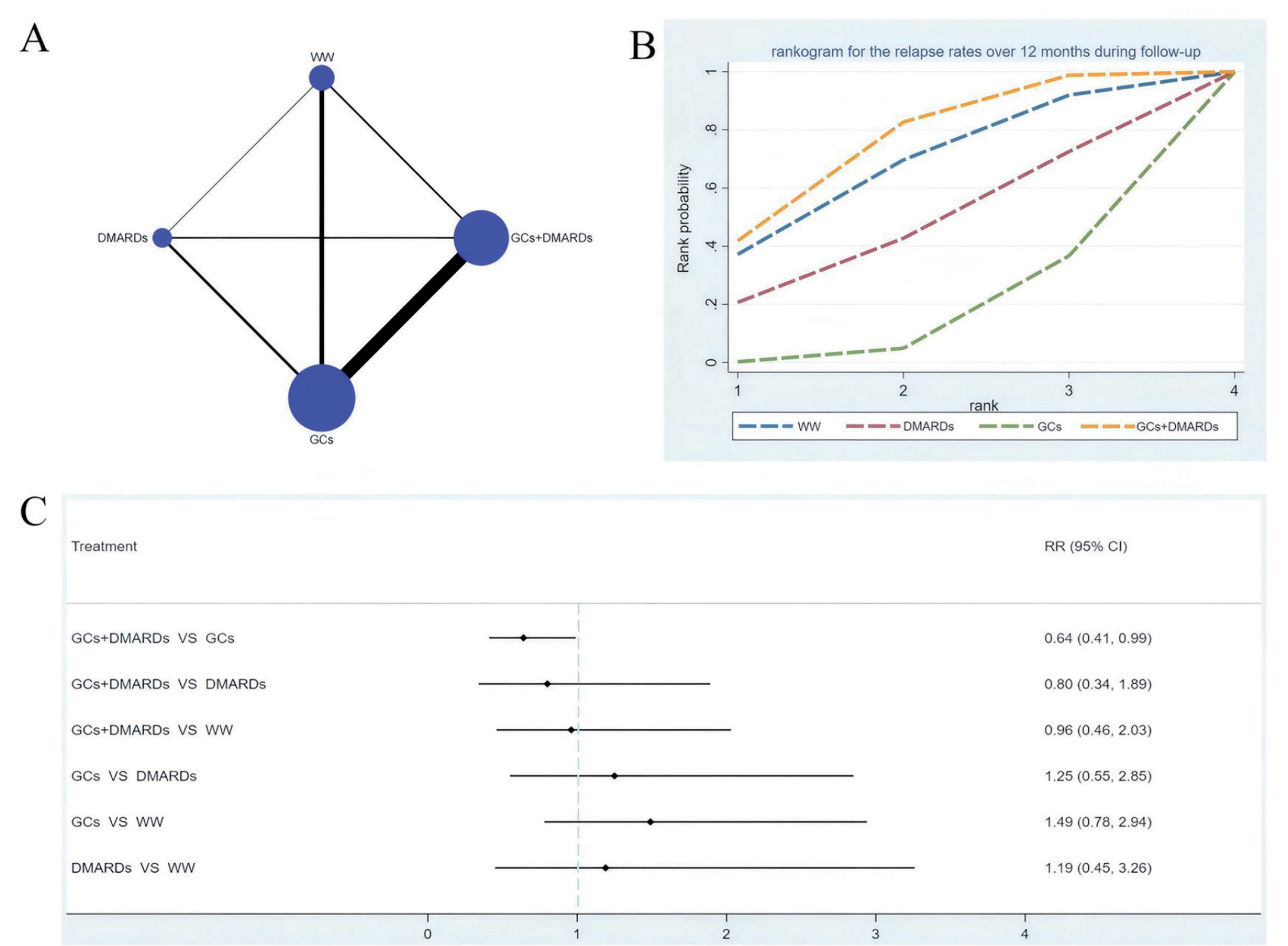

Supplementary Figure 4. Network meta-analysis results for the relapse rates over 12 months. (A): Network diagram; (B): Cumulative probability ranking curve of different interventions; (C): Forest plot. GCs, glucocorticoids; DMARDs, disease-modifying antirheumatic drugs; WW, Watchful waiting.

Supplementary Table 1. PRISMA checklist for network meta-analysis

Section and Topic	Item #	Checklist item	Location where item is reported
**TITLE**			
Title	1	Identify the report as a systematic review.	1
**ABSTRACT**			
Abstract	2	See the PRISMA 2020 for Abstracts checklist.	1
**INTRODUCTION**			
Rationale	3	Describe the rationale for the review in the context of existing knowledge.	1-2
Objectives	4	Provide an explicit statement of the objective(s) or question(s) the review addresses.	1-2
**METHODS**			
Eligibility criteria	5	Specify the inclusion and exclusion criteria for the review and how studies were grouped for the syntheses.	2-5
Information sources	6	Specify all databases, registers, websites, organisations, reference lists and other sources searched or consulted to identify studies. Specify the date when each source was last searched or consulted.	2-5
Search strategy	7	Present the full search strategies for all databases, registers and websites, including any filters and limits used.	2-5
Selection process	8	Specify the methods used to decide whether a study met the inclusion criteria of the review, including how many reviewers screened each record and each report retrieved, whether they worked independently, and if applicable, details of automation tools used in the process.	2-5
Data collection process	9	Specify the methods used to collect data from reports, including how many reviewers collected data from each report, whether they worked independently, any processes for obtaining or confirming data from study investigators, and if applicable, details of automation tools used in the process.	2-5
Data items	10a	List and define all outcomes for which data were sought. Specify whether all results that were compatible with each outcome domain in each study were sought (e.g. for all measures, time points, analyses), and if not, the methods used to decide which results to collect.	2-5
	10b	List and define all other variables for which data were sought (e.g. participant and intervention characteristics, funding sources). Describe any assumptions made about any missing or unclear information.	2-5
Study risk of bias assessment	11	Specify the methods used to assess risk of bias in the included studies, including details of the tool(s) used, how many reviewers assessed each study and whether they worked independently, and if applicable, details of automation tools used in the process.	2-5
Effect measures	12	Specify for each outcome the effect measure(s) (e.g. risk ratio, mean difference) used in the synthesis or presentation of results.	2-5
Synthesis methods	13a	Describe the processes used to decide which studies were eligible for each synthesis (e.g. tabulating the study intervention characteristics and comparing against the planned groups for each synthesis (item #5)).	2-5
	13b	Describe any methods required to prepare the data for presentation or synthesis, such as handling of missing summary statistics, or data conversions.	2-5
	13c	Describe any methods used to tabulate or visually display results of individual studies and syntheses.	2-5
	13d	Describe any methods used to synthesize results and provide a rationale for the choice(s). If meta-analysis was performed, describe the model(s), method(s) to identify the presence and extent of statistical heterogeneity, and software package(s) used.	2-5
	13e	Describe any methods used to explore possible causes of heterogeneity among study results (e.g. subgroup analysis, meta-regression).	2-5
	13f	Describe any sensitivity analyses conducted to assess robustness of the synthesized results.	2-5

(*Continued*)

Supplementary Table 1. PRISMA checklist for network meta-analysis (*Continued*)

Section and Topic	Item #	Checklist item	Location where item is reported
Reporting bias assessment	14	Describe any methods used to assess risk of bias due to missing results in a synthesis (arising from reporting biases).	2-5
Certainty assessment	15	Describe any methods used to assess certainty (or confidence) in the body of evidence for an outcome.	2-5
**RESULTS**			
Study selection	16a	Describe the results of the search and selection process, from the number of records identified in the search to the number of studies included in the review, ideally using a flow diagram.	5-8
	16b	Cite studies that might appear to meet the inclusion criteria, but which were excluded, and explain why they were excluded.	5-8
Study characteristics	17	Cite each included study and present its characteristics.	5-8
Risk of bias in studies	18	Present assessments of risk of bias for each included study.	5-8
Results of individual studies	19	For all outcomes, present, for each study: (a) summary statistics for each group (where appropriate) and (b) an effect estimate and its precision (e.g. confidence/credible interval), ideally using structured tables or plots.	5-8
Results of syntheses	20a	For each synthesis, briefly summarise the characteristics and risk of bias among contributing studies.	5-8
	20b	Present results of all statistical syntheses conducted. If meta-analysis was done, present for each the summary estimate and its precision (e.g. confidence/credible interval) and measures of statistical heterogeneity. If comparing groups, describe the direction of the effect.	5-8
	20c	Present results of all investigations of possible causes of heterogeneity among study results.	5-8
	20d	Present results of all sensitivity analyses conducted to assess the robustness of the synthesized results.	5-8
Reporting biases	21	Present assessments of risk of bias due to missing results (arising from reporting biases) for each synthesis assessed.	5-8
Certainty of evidence	22	Present assessments of certainty (or confidence) in the body of evidence for each outcome assessed.	5-8
**DISCUSSION**			
Discussion	23a	Provide a general interpretation of the results in the context of other evidence.	8-10
	23b	Discuss any limitations of the evidence included in the review.	8-10
	23c	Discuss any limitations of the review processes used.	8-10
	23d	Discuss implications of the results for practice, policy, and future research.	8-10
**OTHER INFORMATION**			
Registration and protocol	24a	Provide registration information for the review, including register name and registration number, or state that the review was not registered.	N/A
	24b	Indicate where the review protocol can be accessed, or state that a protocol was not prepared.	N/A
	24c	Describe and explain any amendments to information provided at registration or in the protocol.	N/A
Support	25	Describe sources of financial or non-financial support for the review, and the role of the funders or sponsors in the review.	Title page
Competing interests	26	Declare any competing interests of review authors.	Title page
Availability of data, code and other materials	27	Report which of the following are publicly available and where they can be found: template data collection forms; data extracted from included studies; data used for all analyses; analytic code; any other materials used in the review.	Title page

From: Page MJ, McKenzie JE, Bossuyt PM, Boutron I, Hoffmann TC, Mulrow CD, et al. The PRISMA 2020 statement: an updated guideline for reporting systematic reviews. BMJ 2021;372:n71. doi: 10.1136/bmj.n71

Supplementary Table 2. Search strategy for network meta-analysis

2.1. Search strategy of Pubmed

No.	Search items
#1	(((((((Immunoglobulin G4-Related Disease[MeSH Terms]) OR (IgG4 Associated Autoimmune Disease*[Title/Abstract])) OR (IgG4 RD[Title/Abstract])) OR (IgG4 Related Disease*[Title/Abstract])) OR (IgG4 related hypertrophic pachymeningitis[Title/Abstract])) OR (IgG4 related pachymeningitis[Title/Abstract])) OR (immunoglobulin G4 related disease*[Title/Abstract])) OR (immunoglobulin G4 related pachymeningitis[Title/Abstract])
#2	((((((((((Glucocorticoids[MeSH Terms]) OR (glucocortico*[Title/Abstract])) OR (glucocortoid[Title/Abstract])) OR (Glucorticoid Effects[Title/Abstract])) OR (Glycocortico*[Title/Abstract])) OR (Prednisone[Title/Abstract])) OR (Prednisolone[Title/Abstract])) OR (Methylprednisolone[Title/Abstract])) OR (Dexamethasone[Title/Abstract])) OR (Hydrocortisone[Title/Abstract])) OR (Cortisone[Title/Abstract])) OR (steroid*[Title/Abstract])
#3	((((((((((((((((((((((((((((((Immunosuppressive Agents[MeSH Terms]) OR (immune suppress*[Title/Abstract])) OR (immunodepressant[Title/Abstract])) OR (Immunosuppress*[Title/Abstract])) OR (Mycophenolate Mofetil[Title/Abstract])) OR (MMF[Title/Abstract])) OR (Azathioprine[Title/Abstract])) OR (AZA[Title/Abstract])) OR (Cyclophosphamide[Title/Abstract])) OR (CYC[Title/Abstract])) OR (Cytoxan[Title/Abstract])) OR (CTX[Title/Abstract])) OR (Leflunomide[Title/Abstract])) OR (LEF[Title/Abstract])) OR (Methotrexate[Title/Abstract])) OR (Amethopterin[Title/Abstract])) OR (MTX[Title/Abstract])) OR (Cyclosporin*[Title/Abstract])) OR (CsA[Title/Abstract])) OR (CyA[Title/Abstract])) OR (Tacrolimus[Title/Abstract])) OR (TAC[Title/Abstract])) OR (iguratimod[Title/Abstract])) OR (IGU[Title/Abstract])) OR (Sulfasalazine[Title/Abstract])) OR (Salicylazosulfapyridine[Title/Abstract])) OR (Sulphasalazine[Title/Abstract])) OR (Salazosulfapyridine[Title/Abstract])) OR (SSZ[Title/Abstract])) OR (Hydroxychloroquine[Title/Abstract])) OR (HCQ[Title/Abstract])) OR (Thalidomide[Title/Abstract])) OR (6-MP[Title/Abstract])
#4	((((((((((((B cell depletion therapies[Title/Abstract]) OR (Rituximab[MeSH Terms])) OR (Rituximab*[Title/Abstract])) OR (Mabthera[Title/Abstract])) OR (Rituxan[Title/Abstract])) OR (RTX[Title/Abstract])) OR (obexelimab[Title/Abstract])) OR (inebilizumab[Title/Abstract])) OR (belimumab[Title/Abstract])) OR (rilzabrutinib[Title/Abstract])) OR (Zanubrutinib[Title/Abstract])) OR (Brukinsa[Title/Abstract])) OR (Bortezomib[Title/Abstract])) OR (Lenalidomide[Title/Abstract])
#5	((((Abatacept[Title/Abstract]) OR (Cytotoxic T Lymphocyte Associated Antigen 4 Immunoglobulin[Title/Abstract])) OR (CTLA4 Ig[Title/Abstract])) OR (CTLA4 immunoglobulin[Title/Abstract])) OR (elotuzumab[Title/Abstract])
#6	(((((dupilumab[Title/Abstract]) OR (Dupixent[Title/Abstract])) OR (mepolizumab[Title/Abstract])) OR (Bosatria[Title/Abstract])) OR (Nucala[Title/Abstract])) OR (tocilizumab[Title/Abstract])
#7	prezalumab[Title/Abstract]
#8	(((((((((Janus Kinase Inhibitors[MeSH Terms]) OR (Janus Kinase Inhibitor*[Title/Abstract])) OR (JAK Inhibitor*[Title/Abstract])) OR (baricitinib[Title/Abstract])) OR (tofacitinib[Title/Abstract])) OR (filgotinib[Title/Abstract])) OR (GLPG0634[Title/Abstract])) OR (upadacitinib[Title/Abstract])) OR (Rinvoq[Title/Abstract])) OR (decernotinib[Title/Abstract])
#9	(((Biological Products[MeSH Terms]) OR (Biologic*[Title/Abstract])) OR (Biopharmaceutical*[Title/Abstract])) OR (Natural Product*[Title/Abstract])
#10	((disease modifying antirheumatic agent[Title/Abstract]) OR (Disease Modifying Antirheumatic Drug*[Title/Abstract])) OR (DMARD*[Title/Abstract])
#11	#2 OR #3 OR #4 OR #5 OR #6 OR #7 OR #8 OR #9 OR #10
#12	#1 AND #11
#13	(((case control[Title/Abstract]) OR (cohort[Title/Abstract])) OR (Retrospect*[Title/Abstract])) OR (Prospect*[Title/Abstract])
#14	#12 AND #13

2.2. Search strategy of Embase

No.	Search items
#1	'immunoglobulin g4 related disease'/exp OR 'igg4 associated autoimmune disease*':ab,ti OR 'igg4 rd':ab,ti OR 'igg4 related disease*':ab,ti OR 'igg4 related hypertrophic pachymeningitis':ab,ti OR 'igg4 related pachymeningitis':ab,ti OR 'immunoglobulin g4 related disease*':ab,ti OR 'immunoglobulin g4 related pachymeningitis':ab,ti
#2	'glucocorticoid'/exp OR glucocortico*:ab,ti OR glucocortoid:ab,ti OR 'glucorticoid effects':ab,ti OR glycocortico*:ab,ti OR prednisone:ab,ti OR prednisolone:ab,ti OR methylprednisolone:ab,ti OR dexamethasone:ab,ti OR hydrocortisone:ab,ti OR cortisone:ab,ti OR steroid*:ab,ti

(*Continued*)

Supplementary Table 2. Search strategy for network meta-analysis (*Continued*)

#3	'immunosuppressive agent'/exp OR 'immune suppress*':ab,ti OR immunodepressant:ab,ti OR immunosuppress*:ab,ti OR 'mycophenolate mofetil':ab,ti OR mmf:ab,ti OR azathioprine:ab,ti OR aza:ab,ti OR cyclophosphamide:ab,ti OR cyc:ab,ti OR cytoxan:ab,ti OR ctx:ab,ti OR leflunomide:ab,ti OR lef:ab,ti OR methotrexate:ab,ti OR amethopterin:ab,ti OR mtx:ab,ti OR cyclosporin*:ab,ti OR csa:ab,ti OR cya:ab,ti OR tacrolimus:ab,ti OR tac:ab,ti OR iguratimod:ab,ti OR igu:ab,ti OR salazosulfapyridine:ab,ti OR salicylazosulfapyridine:ab,ti OR sulphasalazine:ab,ti OR ssz:ab,ti OR hydroxychloroquine:ab,ti OR hcq:ab,ti OR thalidomide:ab,ti OR '6 mp':ab,ti
#4	'b cell depletion therapies':ab,ti OR 'rituximab'/exp OR rituximab*:ab,ti OR mabthera:ab,ti OR rituxan:ab,ti OR rtx:ab,ti OR obexelimab:ab,ti OR inebilizumab:ab,ti OR belimumab:ab,ti OR rilzabrutinib:ab,ti OR zanubrutinib:ab,ti OR brukinsa:ab,ti OR bortezomib:ab,ti OR lenalidomide:ab,ti
#5	abatacept:ab,ti OR 'cytotoxic t lymphocyte associated antigen 4 immunoglobulin':ab,ti OR 'ctla4 ig':ab,ti OR 'ctla4 immunoglobulin':ab,ti OR elotuzumab:ab,ti
#6	dupilumab:ab,ti OR dupixent:ab,ti OR mepolizumab:ab,ti OR bosatria:ab,ti OR nucala:ab,ti OR tocilizumab:ab,ti
#7	prezalumab:ab,ti
#8	'janus kinase inhibitor'/exp OR 'janus kinase inhibitor*':ab,ti OR 'jak inhibitor*':ab,ti OR baricitinib:ab,ti OR tofacitinib:ab,ti OR filgotinib:ab,ti OR glpg0634:ab,ti OR upadacitinib:ab,ti OR rinvoq:ab,ti OR decernotinib:ab,ti
#9	'biological product'/exp OR biologic*:ab,ti OR biopharmaceutical*:ab,ti OR 'natural product*':ab,ti
#10	'disease modifying antirheumatic drug'/exp OR 'disease modifying antirheumatic agent':ab,ti OR 'disease modifying antirheumatic drug*':ab,ti OR dmard*:ab,ti
#11	#2 OR #3 OR #4 OR #5 OR #6 OR #7 OR #8 OR #9 OR #10
#12	#1 AND #11
#13	'case control':ab,ti OR cohort:ab,ti OR retrospect*:ab,ti OR prospect*:ab,ti
#14	#12 AND #13
**2.3**. Search strategy of Cochrane Library	
No.	Search items
#1	MeSH descriptor: [Immunoglobulin G4-Related Disease] explode all trees
#2	(IgG4 Associated Autoimmune Disease*):ti,ab,kw OR (IgG4 RD):ti,ab,kw OR (IgG4 Related Disease*):ti,ab,kw OR (IgG4 related hypertrophic pachymeningitis):ti,ab,kw OR (IgG4 related pachymeningitis):ti,ab,kw OR (immunoglobulin G4 related disease*):ti,ab,kw OR (immunoglobulin G4 related pachymeningitis):ti,ab,kw
#3	#1 AND #2
#4	MeSH descriptor: [Glucocorticoids] explode all trees
#5	(glucocortico*):ti,ab,kw OR (glucocortoid):ti,ab,kw OR (Glucorticoid Effects):ti,ab,kw OR (Glycocortico*):ti,ab,kw OR (Prednisone):ti,ab,kw OR (Prednisolone):ti,ab,kw OR (Methylprednisolone):ti,ab,kw OR (Dexamethasone):ti,ab,kw OR (Hydrocortisone):ti,ab,kw OR (Cortisone):ti,ab,kw OR (steroid*):ti,ab,kw
#6	#4 AND #5
#7	MeSH descriptor: [Immunosuppressive Agents] explode all trees
#8	(immune suppress*):ti,ab,kw OR (immunodepressant):ti,ab,kw OR (Immunosuppress*):ti,ab,kw OR (Mycophenolate Mofetil):ti,ab,kw OR (MMF):ti,ab,kw OR (Azathioprine):ti,ab,kw OR (AZA):ti,ab,kw OR (Cyclophosphamide):ti,ab,kw OR (CYC):ti,ab,kw OR (Cytoxan):ti,ab,kw OR (CTX):ti,ab,kw OR (Leflunomide):ti,ab,kw OR (LEF):ti,ab,kw OR (Methotrexate):ti,ab,kw OR (Amethopterin):ti,ab,kw OR (MTX):ti,ab,kw OR (Cyclosporine):ti,ab,kw OR (Cyclosporin*):ti,ab,kw OR (CsA):ti,ab,kw OR (CyA):ti,ab,kw OR (Tacrolimus):ti,ab,kw OR (TAC):ti,ab,kw OR (iguratimod):ti,ab,kw OR (IGU):ti,ab,kw OR (Sulfasalazine):ti,ab,kw OR (Salicylazosulfapyridine):ti,ab,kw OR (Sulphasalazine):ti,ab,kw OR (Salazosulfapyridine):ti,ab,kw OR (SSZ):ti,ab,kw OR (Hydroxychloroquine):ti,ab,kw OR (HCQ):ti,ab,kw OR (Thalidomide):ti,ab,kw
#9	#7 AND #8
#10	MeSH descriptor: [Rituximab] explode all trees
#11	(B cell depletion therapies):ti,ab,kw OR (Rituximab*):ti,ab,kw OR (Mabthera):ti,ab,kw OR (Rituxan):ti,ab,kw OR (RTX):ti,ab,kw OR (obexelimab):ti,ab,kw OR (inebilizumab):ti,ab,kw OR (belimumab):ti,ab,kw OR (rilzabrutinib):ti,ab,kw OR (Zanubrutinib):ti,ab,kw OR (Brukinsa):ti,ab,kw OR (Bortezomib):ti,ab,kw OR (Lenalidomide):ti,ab,kw
#12	#10 AND #11
#13	(Abatacept):ti,ab,kw OR (Cytotoxic T Lymphocyte Associated Antigen 4 Immunoglobulin):ti,ab,kw OR (CTLA4 Ig):ti,ab,kw OR (CTLA4 immunoglobulin):ti,ab,kw OR (elotuzumab):ti,ab,kw

(*Continued*)

**Supplementary Table 2.** Search strategy for network meta-analysis (*Continued*)	
#14	(dupilumab):ti,ab,kw OR (Dupixent):ti,ab,kw OR (mepolizumab):ti,ab,kw OR (Bosatria):ti,ab,kw OR (Nucala):ti,ab,kw OR (tocilizumab):ti,ab,kw
#15	(prezalumab):ti,ab,kw
#16	MeSH descriptor: [Janus Kinase Inhibitors] explode all trees
#17	(Janus Kinase Inhibitor*):ti,ab,kw OR (JAK Inhibitor*):ti,ab,kw OR (baricitinib):ti,ab,kw OR (tofacitinib):ti,ab,kw OR (GLPG0634):ti,ab,kw OR (filgotinib):ti,ab,kw OR (upadacitinib):ti,ab,kw OR (Rinvoq):ti,ab,kw OR (decernotinib):ti,ab,kw
#18	#16 AND #17
#19	MeSH descriptor: [Biological Products] explode all trees
#20	(Biologic*):ti,ab,kw OR (Biopharmaceutical*):ti,ab,kw OR (Natural Product*):ti,ab,kw
#21	#19 AND #20
#22	(disease modifying antirheumatic drug):ti,ab,kw OR (disease modifying antirheumatic agent):ti,ab,kw OR (Disease Modifying Antirheumatic Drug*):ti,ab,kw OR (DMARD*):ti,ab,kw
#23	#6 AND #9 AND #12 AND #13 AND #14 AND #15 AND #18 AND #21 AND #22
#24	(case control):ti,ab,kw OR (cohort):ti,ab,kw OR (retrospect*):ti,ab,kw OR (prospect*):ti,ab,kw
#25	#3 AND #23 AND #24
**2.4**. Search strategy of Web of Science	
No.	Search items
#1	immunoglobulin G4 related disease (Topic) OR IgG4 Associated Autoimmune Disease* (Topic) OR IgG4 RD (Topic) OR IgG4 Related Disease* (Topic) OR IgG4 related hypertrophic pachymeningitis (Topic) OR IgG4 related pachymeningitis (Topic) OR immunoglobulin G4 related disease* (Topic) OR immunoglobulin G4 related pachymeningitis (Topic)
#2	glucocortico* (Topic) OR glucocortoid (Topic) OR Glucorticoid Effects (Topic) OR Glycocortico* (Topic) OR prednisone (Topic) OR prednisolone (Topic) OR methylprednisolone (Topic) OR dexamethasone (Topic) OR hydrocortisone (Topic) OR cortisone (Topic) OR steroid* (Topic)
#3	immunosuppressive agent (Topic) OR immune suppress* (Topic) OR immunodepressant (Topic) OR Immunosuppress* (Topic) OR mycophenolate mofetil (Topic) OR MMF (Topic) OR azathioprine (Topic) OR AZA (Topic) OR cyclophosphamide (Topic) OR CYC (Topic) OR Cytoxan (Topic) OR CTX (Topic) OR leflunomide (Topic) OR LEF (Topic) OR methotrexate (Topic) OR Amethopterin (Topic) OR MTX (Topic) OR Cyclosporin* (Topic) OR CsA (Topic) OR CyA (Topic) OR tacrolimus (Topic) OR TAC (Topic) OR iguratimod (Topic) OR IGU (Topic) OR Sulfasalazine (Topic) OR salazosulfapyridine (Topic) OR Salicylazosulfapyridine (Topic) OR Sulphasalazine (Topic) OR Salazosulfapyridine (Topic) OR SSZ (Topic) OR hydroxychloroquine (Topic) OR HCQ (Topic) OR thalidomide (Topic) OR 6-MP (Topic)
#4	B cell depletion therapies (Topic) OR Rituximab* (Topic) OR Mabthera (Topic) OR Rituxan (Topic) OR RTX (Topic) OR obexelimab (Topic) OR inebilizumab (Topic) OR belimumab (Topic) OR rilzabrutinib (Topic) OR zanubrutinib (Topic) OR Brukinsa (Topic) OR bortezomib (Topic) OR lenalidomide (Topic)
#5	abatacept (Topic) OR Cytotoxic T Lymphocyte Associated Antigen 4 Immunoglobulin (Topic) OR CTLA4 Ig (Topic) OR CTLA4 immunoglobulin (Topic) OR elotuzumab (Topic)
#6	dupilumab (Topic) OR Dupixent (Topic) OR mepolizumab (Topic) OR Bosatria (Topic) OR Nucala (Topic) OR tocilizumab (Topic)
#7	prezalumab (Topic)
#8	Janus Kinase Inhibitor* (Topic) OR JAK Inhibitor* (Topic) OR baricitinib (Topic) OR tofacitinib (Topic) OR GLPG0634 (Topic) OR filgotinib (Topic) OR upadacitinib (Topic) OR Rinvoq (Topic) OR decernotinib (Topic)
#9	biological product* (Topic) OR Biologic* (Topic) OR Biopharmaceutical* (Topic) OR Natural Product* (Topic)
#10	Disease Modifying Antirheumatic Drug* (Topic) OR disease modifying antirheumatic agent (Topic) OR DMARD* (Topic)
#11	case control (Topic) OR cohort (Topic) OR retrospect* (Topic) OR prospect* (Topic)
#12	#2 OR #3 OR #4 OR #5 OR #6 OR #7 OR #8 OR #9 OR #10
#13	#1 AND #11 AND #12

Supplementary Table 3. Characteristics of the included studies

Author	Year	Country	Age (mean ± SD, years)	Total number	Treatment A	Treatment B	Treatment C	Treatment D	Dose of GCs	Type of DMARDs	Median follow-up time (months)	Main organs involvement	Outcome	NOS score
Ghazale[24]	2008	USA	59.9±15.7	53	GCs	GCs+DMARDs			Started with 40 mg/d for 4 weeks, tapered by 5 mg/wk	AZA, MMF, CTX	6	Cholangitis	②	7
Kubota[25]	2011	Japan	65.9±11.2	70	GCs	WW			NA	NA	46.9	Pancreatitis	②	7
Ebbo[16]	2012	France	56.8±15.0	25	GCs	DMARDs			Started with 0.6mg/kg/d	AZA, CTX, MTX, RTX	60.4	Systemic	①	8
Hart[26]	2012	USA	64.7±14.1	116	GCs	DMARDs	GCs+DMARDs		Started with 40 mg/d for 4 weeks, tapered 5 mg/wk until discontinued	AZA, 6-MP, MMF, RTX	47	Pancreatitis	②	9
Huggett[27]	2014	UK	59.9±13.5	115	GCs	GCs+DMARDs			Started with 30-40 mg/d	AZA, MMF, MTX, 6-MP	33	Pancreatitis and cholangitis	②	7
Carruthers[19]	2015	USA	61.0±11.0	30	DMARDs	GCs+DMARDs			NA	RTX	6	Systemic	①	8
Nada[28]	2016	India	46.6±16.3	10	GCs	GCs+DMARDs			NA	CTX	12	Kidney	①	7
Sekiguchi[6]	2016	USA	59.9±15.7	166	GCs	GCs+DMARDs			Started with 0.4-0.7 mg/kg/d	AZA, 6-MP, MMF, RTX	29	Systemic	②	8
Sun[29]	2016	China	44.8±15.0	17	GCs	GCs+DMARDs			Started with 20-50 mg/d	AZA, CTX, MMF	18	Lung	①②	8
Wallace[30]	2016	USA	55.9±13.2	57	DMARDs	GCs+DMARDs			NA	RTX	8	Systemic	②	7
Chen[31]	2017	China	62.5±16.1	14	GCs	GCs+DMARDs			NA	AZA	23	Pancreatitis	②	7
Karadag[32]	2017	Turkey	51.1±12.7	37	GCs	GCs+DMARDs			Started with 0.5–0.6 mg/kg/d in patients with localized involvement and 1 mg/kg/d in patients with multiorgan involvement	CTX	18	Systemic	①②	7
Yunyun[12]	2017	China	51.6±12.4	102	GCs	GCs+DMARDs			Started with 0.5–1.0mg/kg/d for 1 month, tapered by 5mg per 2 weeks, maintained at 5–10mg/d	CTX	12	Systemic	①②③	9
Fernández-Codina[23]	2018	Spain	55.2±19.7	68	GCs	DMARDs			NA	AZA, MMF	36	Systemic	①②	8
Hong[33]	2018	China	51.8±14.4	43	GCs	GCs+DMARDs			Started with 200 mg/d, followed by 0.6 mg/kg/d, tapered by 2.5-5 mg, maintained at 5 mg/d	CTX, AZA, LEF, MMF	24.6	Salivary	②③	8
Karim[34]	2018	Netherlands	54.7±14.5	32	GCs	DMARDs	GCs+DMARDs		Started with 0.5-1mg/kg/d	CTX, MMF, MTX, AZA, CyA, RTX, thalidomide	12	Systemic	②	8
Park[35]	2018	Korea	55.2(Median)	42	GCs	GCs+DMARDs			Started with 0.6–0.8mg/kg/d, tapered by 10mg every 2–4 weeks	AZA, MTX, CyA	24	Ophthalmic	②	7
Wallwork[36]	2018	USA	53.8±3.8	26	DMARDs	GCs+DMARDs			Started with 20– 80mg/day	RTX	6	Retroperitoneal fibrosis	③	7
Wang[37]	2018	China	53.5±13.4	215	GCs	GCs+DMARDs			Started with 0.5-1.0 mg/kg/d, tapered by 5 mg, maintained at 5-10 mg/d	CTX, MMF, MTX, AZA, LEF, CyA, T2	6	Systemic	①②③	9
Xin[38]	2018	China	59.0±11.1	101	GCs	GCs+DMARDs			Started with 0.6 mg/kg/d, tapered by 5 mg every 1 to 2 weeks	AZA, MMF	40	Pancreatitis	②	8
Fei[11]	2019	China	56.0±12.2	69	GCs	GCs+DMARDs			Started with 0.6–0.8 mg/kg/d for 1 month, tapered by 5mg per 2 weeks, maintained at 7.5–10mg/d	MMF	12	Systemic	①②③	9

(*Continued*)

Supplementary Table 3. Characteristics of the included studies (*Continued*)

Author	Year	Country	Age (mean ± SD, years)	Total number	Treatment A	Treatment B	Treatment C	Treatment D	Dose of GCs	Type of DMARDs	Median follow-up time (months)	Main organs involvement	Outcome	NOS score
Peng[39]	2019	China	51.9±13.3	122	GCs	GCs+DMARDs			Started with 0.6-1.0 mg/kg/d, tapered by 5 mg per 1 or 2 weeks	CTX, MMF, MTX, AZA, LEF, CyA	12	Systemic	②	9
Liu[40]	2020	China	57.9±2.3	277	GCs	GCs+DMARDs			Started with 0.6-0.8 mg/kg/d, tapered by 5 mg, maintained at 5-10 mg/d	CTX, MMF, MTX, AZA, LEF	21	Systemic	②	9
Phaopraphat[41]	2020	Thailand	59.6±13.3	110	GCs	GCs+DMARDs			NA	CTX, MMF, MTX, AZA, RTX	22.2	Systemic	②	7
Gan[15]	2021	China	51.7±11.1	132	GCs	DMARDs	GCs+DMARDs	WW	Started with 0.6-1.0 mg/kg/d, tapered by 5 mg, maintained at ≤10 mg/d	CTX, MMF, MTX	6	Ophthalmic	①②	8
Peng[42]	2021	China	53.8±12.5	143	GCs	GCs+DMARDs			Started with 0.5-1.0 mg/kg/d	CTX, MMF, MTX, LEF	24	Systemic	②	8
Zongfei[43]	2021	China	58.7±3.0	102	GCs	GCs+DMARDs	WW		Started with 0.6-1.0 mg/kg/d, gradually tapered to a maintenance dose of 0.1–0.2 mg/kg/d	CTX, AZA, RTX	21	Systemic	①②	8
An[44]	2022	China	62.0±12.8	92	GCs	WW			NA	NA	11	Systemic	①	8
Kubota[45]	2022	Japan	NA	896	GCs	WW			Started with 0.6 mg/kg/d, maintained at 5–10mg/d	NA	45	Cholangitis	①②	7
Matza[9]	2022	USA	648±12.2	10	DMARDs	GCs+DMARDs			NA	Abatacept	6	Systemic	①	7
Son[46]	2022	Korea	50.9±9.2	38	GCs	GCs+DMARDs			NA	MTX, CyA, AZA	33.6	Ophthalmic	①	7
Chaba[47]	2023	France, Belgium	67.8±3.8	101	GCs	GCs+DMARDs			Started with a mean dose of 0.8±0.3 mg/kg/d	RTX	12	Kidney	②③	8
Overbeek[48]	2023	Europe	57.0±20.0	735	GCs	DMARDs	WW		Started with 40 mg/d	AZA, MTX, RTX	30	Pancreatitis	①②	8
Zhang[14]	2023	China	57.6±12.8	171	GCs	GCs+DMARDs			Started with 30–40 mg/d for 1 month, tapered by 5mg per 1 or 2 weeks, maintained at 2.5–5.0mg/d	CTX, MMF, MTX, TAC, triptolide	12	Systemic	①②	9

Note: GCs, glucocorticoids; DMARDs, disease-modifying antirheumatic drugs; WW, Watchful waiting; AZA, azathioprine; CTX, cyclophosphamide; MMF, mycophenolate mofetil; MTX, methotrexate; LEF, leflunomide; RTX, Rituximab; TAC, tacrolimus; CyA, cyclosporine A; T2, tripterysium glycosides; 6-MP, 6-mercaptopurine; NA, not available; ①, remission; ②, relapse; ③, adverse reactions.

Supplementary Table 4. Qualitative analysis of adverse events by system organ class

Organ System	Adverse Event	GCs+DMARDs (n=285)	DMARDs (n=19)	GCs (n=252)
Gastrointestinal	Gastrointestinal reactions	23 (8.1%)	0 (0%)	12 (4.8%)
Liver	Impaired hepatic function	6 (2.1%)	0 (0%)	6 (2.4%)
Metabolic	Glucose intolerance	31 (10.9%)	0 (0%)	25 (9.9%)
Infection	Infections	43 (15.1%)	1 (5.3%)	38 (15.1%)
Cardiovascular	Hypertension	2 (0.7%)	0 (0%)	3 (1.2%)
Hematological	Leukopenia/Myelosuppression	6 (2.1%)	0 (0%)	0 (0%)

Supplementary Table 5. Organ involvement in different regional cohorts

Affecting organ or tissue	Asian cohorts (n=1634)	Non-Asian cohorts (n=440)
Lymph gland	681(41.68%)	109(24.77%)
Salivary glands	677(41.43%)	105(23.86%)
Pancreas	587(35.92%)	179(40.68%)
Bile duct	466(28.52%)	126(28.64%)
Lacrimal gland	395(24.17%)	47(10.68%)
Lung/pleura	386(23.62%)	66(15.00%)
Orbit	287(17.56%)	73(16.59%)
Retroperitoneal fibrosis	269(16.46%)	91(20.68%)
Nose	243(14.87%)	38(8.64%)
Kidney	173(10.59%)	60(13.64%)
Prostate	125(7.65%)	9(2.05%)
Aorta	69(4.22%)	47(10.68%)
Thyroid	58(3.55%)	12(2.73%)
Liver	29(1.77%)	23(5.23%)
Mediastina	26(1.59%)	10(2.27%)
Pituitary	12(0.73%)	4(0.91%)
Mater	12(0.73%)	6(1.36%)
Skin	12(0.73%)	12(2.73%)
Gastrointestinal tract	10(0.61%)	12(2.73%)
Ear	3(0.18%)	4(0.91%)
Pericardium	1(0.06%)	13(2.95%)
Breast	1(0.06%)	2(0.45%)

Supplementary Table 6. Subgroup analysis in a network frame by region

A			
GCs+DMARDs	RR(95% CI)		
1.15 (0.95, 1.39)	GCs		
0.99 (0.62, 1.69)	0.86 (0.54, 1.47)	DMARDs	
**1.83 (1.26, 2.62)**	**1.6 (1.13, 2.21)**	1.85 (1, 3.17)	WW
B			
GCs+DMARDs	RR(95% CI)		
**0.62 (0.44, 0.85)**	GCs		
0.61 (0.22, 1.68)	0.99 (0.37, 2.74)	DMARDs	
0.64 (0.35, 1.22)	1.04 (0.6, 1.91)	1.05(0.36,3.11)	WW
C			
GCs+DMARDs	RR(95% CI)		
1.32 (0.59, 3.99)	GCs		
1.33 (0.66, 3.77)	0.99 (0.67, 1.47)	DMARDs	
D			
GCs+DMARDs	RR(95% CI)		
**0.54 (0.33, 0.87)**	GCs		
0.65 (0.31, 1.48)	1.2 (0.59, 2.84)	DMARDs	

Note: (A): the overall remission rates in Asian patients; (B): the overall relapse rates in Asian patients; (C): the overall remission rates in Non-Asian patients; (D): the overall relapse rates in Non-Asian patients. The cells contain the RR (95% CI) of the medication protocol on the left compared to the medication protocol on the right. Bolded values are statistically significant. GCs, glucocorticoids; DMARDs, disease-modifying antirheumatic drugs; WW, Watchful waiting.

Supplementary Table 7. Subgroup analysis in a network frame by different DMARDs

A					
GCs+cDMARDs	RR(95% CI)				
1.05 (0.61, 1.89)	GCs+bDMARDs				
1.20 (0.85, 1.74)	1.14 (0.59, 2.17)	cDMARDs			
0.85 (0.57, 1.29)	0.81 (0.41, 1.53)	0.71 (0.49, 1.03)	bDMARDs		
1.15 (0.94, 1.41)	1.09 (0.59, 1.93)	0.95 (0.68, 1.30)	1.34 (0.92, 1.93)	GCs	
**1.72 (1.26, 2.39)**	1.63 (0.85, 3.07)	1.43 (0.94, 2.15)	**2.01 (1.28, 3.18)**	**1.50 (1.14, 2.00)**	WW
B					
GCs+cDMARDs	RR(95% CI)				
1.47 (0.44, 5.62)	GCs+bDMARDs				
0.63 (0.30, 1.40)	0.43 (0.11, 1.58)	cDMARDs			
1.11 (0.33, 4.46)	0.76 (0.23, 2.49)	1.74 (0.53, 6.80)	bDMARDs		
**0.59 (0.43, 0.80)**	0.40 (0.11, 1.29)	0.93 (0.43, 1.90)	0.53 (0.13, 1.73)	GCs	
0.88 (0.47, 1.66)	0.6 0(0.14, 2.22)	1.38 (0.56, 3.38)	0.79 (0.18, 2.96)	1.50 (0.85, 2.71)	WW

Note: (A): the overall remission rates between different medication protocols; (B): the overall relapse rates between different medication protocols; The cells contain the RR (95% CI) of the medication protocol on the left compared to the medication protocol on the right. Bolded values are statistically significant. GCs, glucocorticoids; cDMARDs, conventional disease-modifying antirheumatic drugs; bDMARDs, biological conventional disease-modifying antirheumatic drugs; WW, Watchful waiting
